# Transforaminal Interbody Impaction of Bone Graft to Treat Collapsed Nonhealed Vertebral Fractures with Endplate Destruction: A Report of Two Cases

**DOI:** 10.1155/2020/8873350

**Published:** 2020-09-02

**Authors:** Adam M. Wegner, Yu-Hsuan Chou, Hsiao-Kang Chang, Tsung-Cheng Yin

**Affiliations:** ^1^OrthoCarolina, Winston-Salem, North Carolina, USA; ^2^Changhua Christian Hospital, Changhua, Taiwan; ^3^Department of Orthopedic Surgery, Kaohsiung Chang Gung Memorial Hospital, Kaohsiung, Taiwan

## Abstract

**Background:**

A collapsed nonhealed vertebral fracture with endplate destruction is a challenging injury to address, as there is no single definitive treatment. We present two cases using an innovative transforaminal grafting technique to treat these patients. *Case Presentation*. Case 1: a 72-year-old woman had nonunion of an L1 compression fracture with destruction of both endplates. T12/L1 and L1/L2 transforaminal debridement and impaction of bone graft were performed followed by posterior instrumentation. At three years follow-up, the fusion mass between T12/L1 and L1/L2 was solid and the patient had minimal pain. Case 2: a 62-year-old woman had nonunion of an L1 burst fracture with destruction of the lower endplate. Hemilaminectomy and transforaminal interbody impaction of bone graft was performed. At three years follow-up, the patient had no back pain and a solid fusion. In both cases, local kyphosis was corrected and fusion obtained.

**Conclusions:**

Collapsed nonhealed vertebral body fractures combined with endplate destruction can be successfully treated with a one-step posterior surgery consisting of transforaminal debridement and impaction of bone graft in combination with posterior pedicle instrumentation.

## 1. Background

A collapsed nonhealed vertebral fracture with endplate destruction is a challenging problem [1], as there is no single definitive treatment for this type of injury. Kyphoplasty or vertebroplasty risk cement leakage, recurrent vertebral collapse, and bone cement dislodgement due to endplate destruction [2, 3]. Anterior approaches for vertebral body reconstruction with allograft or metal cages at the thoracolumbar junction may provide reliable support [4], but they are time-consuming and associated with higher morbidity than posterior approaches [5]. Although the transpedicular bone grafting technique was developed for burst fractures [6, 7], it is not uncommon for interbody fusions to fail after these procedures [8]. Using the transpedicular route, the surgeon may find it difficult to prepare the disc space and the adjacent endplates well, both important steps to achieve interbody fusion. However, if the transforaminal route is taken, the disc space can be more easily prepared, and bone graft can be directly impacted into the void created by endplate destruction and disc space, resulting in better anterior support and interbody fusion. We present two cases of collapsed nonhealed vertebral fractures with endplate destruction at the thoracolumbar junction that were treated using transforaminal interbody impaction of allograft. This the first report of this technique to treat collapsed nonhealed vertebral fractures with endplate destruction.

## 2. Case Presentations

**Figure 1:**
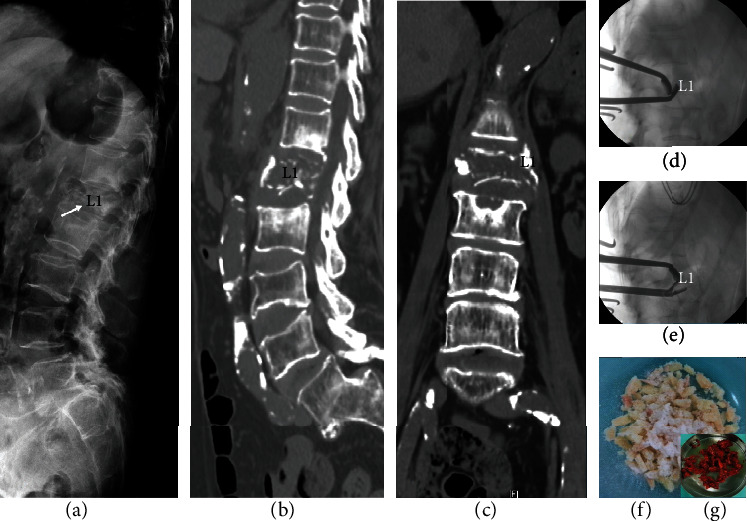
(a) Lateral radiograph and (b) axial and (c) coronal CT reconstructions showed a collapsed nonhealing L1 vertebral body with destruction of both upper and lower endplates. (d, e) T11/T12 and T12/L1 transforaminal debridement using curettes to remove disc material and cartilage. (f) Antibiotic impregnated allograft. (g) Autologous bone graft from posterior iliac crest.

**Figure 2:**
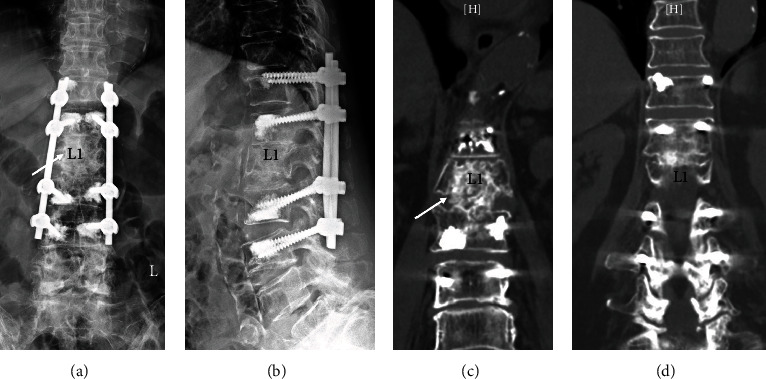
(a, b) After transforaminal debridement and impaction of bone graft, posterior pedicle instrumentation using cement-augmented pedicle screws was placed from T11-L3. At three years follow-up, the fusion mass between L1/L2 (c) and T12/L1 (d) was solid.

Case 1 involved a 72-year-old female with underlying diabetes mellitus, hypertension, and prior cerebral vascular accident (CVA). The patient had been previously treated for T12/L1 and L1/L2 pyogenic discitis with concurrent L1 vertebral osteomyelitis with antibiotics 1 year prior. She was referred to our clinic due to progressive back pain. She had completed her course of antibiotics four months prior, and C-reactive protein (CRP) had been below 5 mg/L for 2 months. Radiographs and CT showed a collapsed nonhealing L1 vertebra with destruction of both upper and lower endplates (Figures 1(a)–1(c)). Due to the patient's age and underlying comorbidities, we wanted to avoid an anterior approach, so a posterior one-stage approach was used. T12/L1 and L1/L2 transforaminal debridement and impaction of mixed autologous bone and allograft (Figures 1(f) and 1(g)) were performed for T12-L1-L2 interbody fusion (Figures 1(d) and 1(e)), followed by posterior instrumentation using cement-augmented pedicle screws from T11-L3 (Figures 2(a) and 2(b)). Postoperatively, back pain improved significantly. Because the patient had osteoporosis (BMD T-score: -2.8), the patient was given teriparatide 20 *μ*g daily for 12 months after surgery to promote growth of new bone. After 12 months of teriparatide treatment, the patient was switched to 60 mg denosumab every 6 months, which they are still currently taking. At three years follow-up, the fusion mass between T12/L1 and L1/L2 was solid, and the patient had minimal pain (Figures 2(c) and 2(d)). Instrumentation has remained intact without loosening until the latest follow-up, and no further surgery to remove instrumentation is planned.

**Figure 3:**
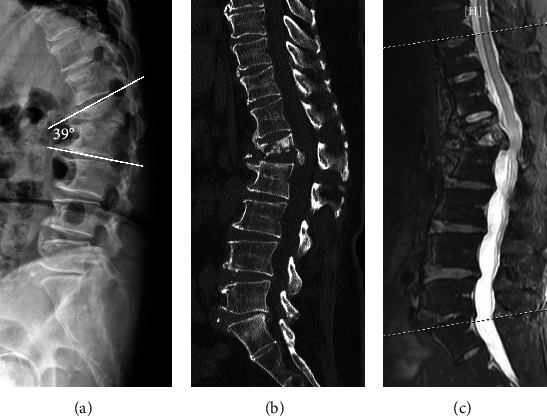
(a) Lateral radiographs showed a collapsed nonhealed L1 fracture and local kyphosis (39 degrees). Sagittal CT (b) and MRI (c) show a retropulsed bone fragment which compromised the spinal canal.

**Figure 4:**
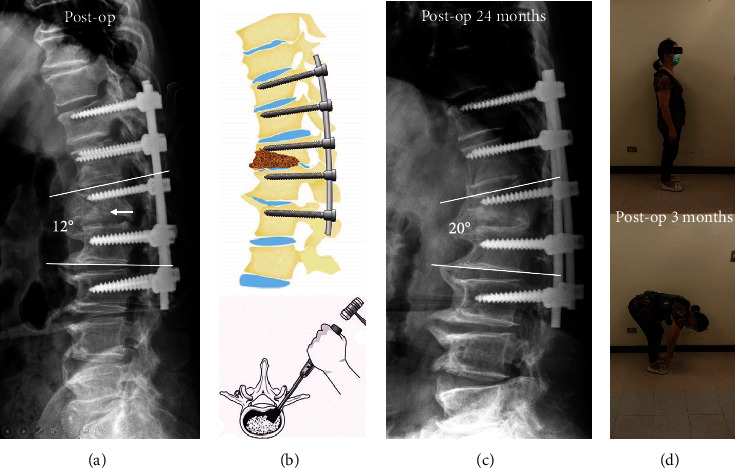
(a) Postoperative kyphotic angle improved immediately post operatively from 39 degrees to 12 degrees. (b) Allograft was impacted at the L1/T12 disc level. (c) The kyphotic angle stabilized at 20 degrees at three years follow-up. (d) At three months follow-up, back pain VAS had improved from 8 to 1 and the patient was able to bend forward without notable pain.

Case 2 involved a 62-year-old woman who had received conservative treatment for an L1 burst fracture six months prior. At that time, the patient developed progressive back pain and kyphotic deformity, for which she was referred to our clinic. Radiographs showed a collapsed nonhealed L1 fracture and 39 degrees of local kyphosis (Figure 3(a)). CT and MRI showed L1 vertebral body destruction similar to Kummell's disease, with destruction of the inferior endplate (Figures 3(b) and 3(c)). A retropulsed bone fragment was observed in the canal (Figure 3(c)). The patient also complained of left inguinal pain. Left hemilaminectomy was performed at T12/L1, followed by transforaminal interbody impaction of allograft into the T12/L1 disc space (Figure 4(b)). The postoperative kyphotic angle improved from 39 degrees to 12 degrees (Figure 4(a)). At three months follow-up, her VAS back pain had improved from 8 to 1, and she was able to bend forward without pain (Figure 4(c)). Due to osteoporosis (BMD: T-score: -2.6), teriparatide 20 *μ*g daily was given for 3 months, followed by denosumab 60 mg every 6 months, which the patient continues to take. The kyphotic angle was 20 degrees at three years follow-up (Figure 4(d)), at which time X-ray showed solid union and instrumentation remained intact. The patient has no back pain and has kept regular radiographic and clinical follow-up to 3 years.

## 3. Surgical Approach and Procedures

The surgical approach was the same as a standard open transforaminal lumbar interbody fusion (TLIF) procedure. First, autogenous iliac crest bone graft was harvested. This is due to the fact that bone morphogenetic protein (BMP) is not available for use in our facility; therefore, autograft and allograft were combined for bone grafting. Posterior iliac crest provides large quantities of bone graft and is accessible during posterior spine procedures. It is osteogenesic, osteoinductive, and osteoconductive.

The procedure for posterior iliac bone harvest was as follows. After exposure of the posterior iliac crest, the outer surface was visualized with the use of a Taylor retractor. Caution was taken to avoid penetrating the sciatic notch and potentially injuring the superior gluteal artery. Using a straight osteotome, multiple corticocancellous vertical strips were cut from the iliac crest edge. A curved osteotome was used to complete the cuts distally. After removal of the corticocancellous strips, gouges or curettes were used to harvest additional cancellous bone (Figure 1(g)).

Since the TLIFs were performed at the thoracolumbar junction, dural retraction was absolutely avoided to minimize trauma to the spinal cord. A total facetectomy was performed on the side of the patient's radicular symptoms. In case 2, the patient had left inguinal pain, so a left-sided TLIF approach was performed, consisting of left total facetectomy and decompression of the left L1 exiting nerve root. If the patient had bilateral radiculopathy or spinal cord compression, we would have performed bilateral hemilaminectomy to decompress the nerve roots or dura along with a unilateral total facetectomy to performed discectomy and grafting. In both cases, there was minimal anterior spinal column support due to bony destruction, so posterior stability was maintained by preserving the spinous processes, supraspinous ligaments, interspinous ligaments, and the opposite side facet joint if possible. After adequate decompression of the neural elements, pedicle screws were placed. The disc space was gradually distracted using the pedicle screws or a laminar spreader. After exposure of the posterior annulus, a complete discectomy was performed using disc rongeurs, disc shavers, and curved curettes. The disc and cartilage endplate must be completely removed to provide a local environment conducive to fusion. It should be noted that the outer layer of annulus fibrosis should be kept intact to contain bone graft within the disc space, so it does not extrude out anteriorly. After placement of bone graft, a cage implant trial was used as a bone impactor. The bone void and disc space were solidly packed with graft after 3 or 4 rounds of graft impaction. After verifying bone graft and screw position with fluoroscopy, 5.5 mm titanium rods were placed to complete the procedure.

## 4. Discussion and Conclusions

Osteonecrosis of the vertebral body, also known as Kummell's disease, is a challenging condition for spine surgeons to manage [9]. Vertebroplasty and kyphoplasty can be used to achieve early pain control by stabilizing the intravertebral cleft but do not correct the kyphotic deformity well. The collapsed nonhealed fractures in these two cases were just like the Kummell's disease, in that both patients had destruction of the vertebral body as well as endplate injury. Based on the radiographs, the bony destruction was so severe that it violated the end-plate. Under these circumstances, it would be difficult to recover anterior column height by vertebroplasty or kyphoplasty, and there would be risk of cement extravasation [10].

A metallic implant was not chosen for anterior reconstruction because more posterior structures need to be sacrificed to place the metallic implant in the anterior column from a posterior approach. The impaction of cancellous bone chips for anterior reconstruction through the transforaminal route preserves more posterior structure and shortens the surgical time. In case 1, there was destruction of the L2 upper endplate, so there was a risk of subsidence of a metallic implant into the L2 vertebral body.

Therefore, we decided to perform vertebral body reconstruction using posterior transforaminal impaction bone grafting.

By impacting bone graft directly into the bone defect and intervertebral space, we could achieve additional immediate stability of the anterior and middle columns. It should also be noted that allograft offers more bone volume than autograft and is more bioavailable and fills the void more effectively compared to metal mesh or cages [11, 12]. This technique had been introduced to treat the infectious spondylodiscitis [13], but there are no reports describing its use in the treatment of collapsed nonhealed vertebral fractures with endplate destruction. Posterior pedicle instrumentation is one of the most reliable surgical treatments for thoracolumbar fractures, because it grants rigid fixation. However, determining the number of levels to instrument above and below the fracture to achieve a successful recovery is still controversial [14–16]. In our two cases, even with impacted the bone graft for anterior column support, we decided to instrument two vertebrae above and two vertebrae below the injury level to provide enough stabilization to enable early mobilization. Although long segment pedicle screw fixation preserves fewer motion segments in the lumbar spine, for our two cases, bony union and fusion was achieved at the fracture site. No implant failure or loosening necessitating revision occurred, even though there was an increase of kyphotic angle (12 degrees to 20 degrees). In both cases, the low instrumented vertebral was L3, which did not significantly affect lumbar lordosis postoperatively, as a majority of lumbar lordosis is derived from the low lumbar region.

The transpedicular bone grafting technique was developed for burst fractures [17, 18], but it is not uncommon for interbody fusions to fail after these procedures [8]. For case 1, there was no bony fragment behind the vertebral body; for case 2, the retropulsed bone fragment was at the lower corner, which is close to disc space level. Using the transpedicular route, the surgeon may find it difficult to prepare the disc space and adjacent endplates well, both important steps to improve interbody fusion. However, if the transforaminal route is taken, the disc space can be more easily prepared, and bone graft can be directly impacted into the void created by endplate destruction and disc space, resulting in better anterior support and interbody fusion.

Both patients were osteoporotic, so they received postoperative medication to improve bone density. We believed that the secondary prevention of osteoporotic vertebral fracture was critical and should be emphasized [19].

This is the first report of transforaminal impaction of bone graft to treat collapsed nonhealed vertebral body fractures with endplate destruction. Surgical outcomes were satisfactory at 3 years follow-up. We believe that transforaminal impaction of bone graft impaction in combination with posterior pedicle instrumentation is a simple viable alternative option to treat this very difficult problem. However, further study is required to compare advantages and disadvantages of this innovative surgical technique.

## Abbreviations

CRP: C-reactive protein
TLIF: Transforaminal lumbar interbody fusion
BMD: Bone mineral density
CT: Computed tomography
MRI: Magnetic resonance imaging.
